# Evaluation of the Miscibility of Novel Cocoa Butter Equivalents by Raman Mapping and Multivariate Curve Resolution–Alternating Least Squares

**DOI:** 10.3390/foods10123101

**Published:** 2021-12-14

**Authors:** Efraín M. Castro-Alayo, Llisela Torrejón-Valqui, Ilse S. Cayo-Colca, Fiorella P. Cárdenas-Toro

**Affiliations:** 1 Facultad de Ingeniería y Ciencias Agrarias, Instituto de Investigación, Innovación y Desarrollo para el Sector Agrario y Agroindustrial de la Región Amazonas (IIDAA), Universidad Nacional Toribio Rodríguez de Mendoza de Amazonas, Calle Higos Urco 342-350-356, Chachapoyas 01001, Peru; llisela.torrejon@untrm.edu.pe; 2 Sección de Ingeniería Industrial, Departamento de Ingeniería, Pontificia Universidad Católica del Perú, Av. Universitaria 1801, San Miguel 15088, Peru; fcardenas@pucp.pe; 3 Programa de Doctorado en Ingeniería, Departamento de Ingeniería, Pontificia Universidad Católica del Perú, Av. Universitaria 1801, San Miguel 15088, Peru; 4 Facultad de Ingeniería Zootecnista, Agronegocios y Biotecnología, Universidad Nacional Toribio Rodríguez de Mendoza de Amazonas, Calle Higos Urco 342-350-356, Chachapoyas 01001, Peru; icayo.fizab@untrm.edu.pe

**Keywords:** cocoa butter, coconut oil, sacha inchi oil, confocal raman microscopy, raman mapping, multivariate curve resolution–alternating least squares, chocolate

## Abstract

Cocoa butter (CB) is an ingredient traditionally used in the manufacturing of chocolates, but its availability is decreasing due to its scarcity and high cost. For this reason, other vegetable oils, known as cocoa butter equivalents (CBE), are used to replace CB partially or wholly. In the present work, two Peruvian vegetable oils, coconut oil (CNO) and sacha inchi oil (SIO), are proposed as novel CBEs. Confocal Raman microscopy (CRM) was used for the chemical differentiation and polymorphism of these oils with CB based on their Raman spectra. To analyze their miscibility, two types of blends were prepared: CB with CNO, and CB with SIO. Both were prepared at 5 different concentrations (5%, 15%, 25%, 35%, and 45%). Raman mapping was used to obtain the chemical maps of the blends and analyze their miscibility through distribution maps, histograms and relative standard deviation (RSD). These values were obtained with multivariate curve resolution–alternating least squares. The results show that both vegetable oils are miscible with CB at high concentrations: 45% for CNO and 35% for SIO. At low concentrations, their miscibility decreases. This shows that it is possible to consider these vegetable oils as novel CBEs in the manufacturing of chocolates.

## 1. Introduction

In the manufacture of chocolates, one of the main ingredients is cocoa butter (CB), which is the main contributor to the high fat content of chocolate. In total, 30 to 40% of the weight of chocolate is fat [1]. CB remains as a solid at 20 °C (with hard texture and snap) and melts rapidly at 33 °C, leading to the release of flavor and soft mouth-feel texture [2,3]. Cost and technical limitations have increased CB demand, although it is the main lipid phase in chocolate manufacturing. In addition, it is also used in combination with other vegetable oils, such as hydrogenated or partially hydrogenated soybean oil and palm oil [2,3,4]. Therefore, researchers have been searching for cheaper alternatives with similar characteristics to CB [2]. These alternatives should improve the physical properties and bloom resistance and reduce the health risks of the final product [3]. CB can be substituted with vegetable fats by blending to produce chocolate or replace CB either partially or wholly [2]. These are the so-called cocoa butter equivalents (CBE), which should be compatible with CB without presenting any eutectic behavior [5]. According to Norazlina et al. [2], CBEs are nonlauric fats that are obtained from the fractionation, interesterification and blending of fats and oils. CBEs have physicochemical, thermal and sensory attributes similar and compatible with those of CB, so they can be miscible in any proportion without changing the characteristics of CB (that is, they are fully compatible with CB properties) [2,6,7,8]. CBEs also possess TAGs similar to those in CB but are produced from low-cost vegetable oils [9]. Some countries allow the use of noncocoa vegetable fats or oils at a defined maximum level to improve the properties of chocolate [10]. The current legislation of the European Union allows the incorporation of up to 5% of CBE in the total weight of the chocolate [11], while the United States legislation does not specify this value [12,13,14]. European regulation only allows six vegetable oils to be used as CBE, specifically illipe, palm oil, sal, shea, kokum gurgi and mango kernel [11,13].

In the Peruvian amazon, sacha inchi (*Plukenetia huayabambana* L.) is cultivated. This plant is known as the Inca peanut and is an important source of phenolic compounds and high antioxidant capacity [15,16]. Sacha inchi oil (SIO) is emerging as a functional food due to its rich composition of polyunsaturated fatty acids, tocopherol and sterols. These compounds have shown multiple human health benefits [17]. Coconut oil (CNO) is used in food manufacturing. It is a saturated fat rich in small and medium chain fatty acids, comparable to animal fat [18]. The scientific and nonspecialized literature promotes the consumption of CNO based on the assumption that it is beneficial for health because it is low in cholesterol, reduces the risk of cardiovascular diseases, encourages weight loss, and improves cognitive functions, among others [19]. In Peru, these natural vegetable oils are produced, and could be good candidates to be considered novel CBEs.

During food manufacturing, some ingredients, even when they may be macroscopically miscible, can show microscopic heterogeneities that would cause instability and phase separation during storage [20]. This is a problem in the manufacture of pharmaceutical tablets, but is also a problem in the manufacture of chocolates. To ensure the quality of the final product, analytical techniques are used to create chemical maps [21] and determinate their miscibility. Some researchers are developing methodologies based on Raman mapping (or Raman imaging) for the evaluation of miscibility between ingredients [20,22,23,24]. Raman spectroscopy can offer widespread food safety assessments in a nondestructive, easy to operate, sensitive, and rapid manner [25]. Raman mapping assimilates two important technologies, imaging and Raman spectroscopy, to simultaneously provide an image of, and spectral information regarding, food products [26]. The purpose of Raman mapping is to visualize the distribution of components by chemical properties in a sample [25] and to study heterogeneous materials, since it provides submicron spatial resolution with high sensitivity [27].

In Raman mapping, each pixel in the image corresponds to a Raman spectrum, which is compared to an established Raman database to determine the specific analytes or spectral background measurements in this location [25]. The spectral information obtained is complex, so multivariate analysis is necessary to unravel complex spectral data from Raman mapping data [21]. Multivariate curve resolution–alternating least squares (MCR–ALS) is a self-modeling curve resolution method that offers the possibility of extracting physically meaningful spectra associated with pure components from the mixed Raman spectra of real biological samples with the benefit of not requiring prior information about the nature of the sample [28]. Using MCR–ALS, Mitsutake et al. [22] found that CB and CNO present intermediate miscibility at concentrations of 75% and 25%, respectively, when used in the manufacture of pharmaceutical tablets.

Raman mapping is expected to be a useful tool for the food industry to assess the quality and safety of food [26]; however, there is scarce information on this topic. The present work focuses on two important approaches for the food industry: the search for natural sources of Peruvian origin for use in the chocolate industry as novel CBEs, and the use of a new non-destructive analysis technique to evaluate the miscibility of these natural sources with CB as a first step for the development of new CBEs. Thus, the objective of this work was to study the miscibility of CNO, SIO, and CB using confocal Raman microscopy and MCR–ALS to propose novel CBEs for the chocolate industry.

## 2. Materials and Methods

### 2.1. Materials

The vegetable oils used were pure CNO and SIO, which were purchased from a local market in Chachapoyas, Peru. CB was provided by the Cooperativa de Servicios Múltiples Aprocam (Bagua, Amazonas, Peru).

### 2.2. Sample Preparation

Following Mitsutake et al. [22], the samples were prepared by heating them to 10 °C above the CB melting point; the materials were added while stirring until a visually homogeneous blend was obtained. Two batches were prepared: the first was composed of CB and CNO (CB-CNO), and the second was composed of CB and SIO (CB-SIO). Each batch contained 5 concentrations of vegetable oils, ranging from 5% to 45% (Table 1), and 3 replicates of each concentration were produced. The samples were placed in a chocolate mold and cooled to 4 °C for easy removal of the tablets. A piece of 1 × 1 cm^2^ was removed from each tablet for Raman mapping.

### 2.3. Raman Mapping

Following the process of Mitsutake et al. [22] with some modifications, the samples were mapped using a Raman confocal microscope system (Horiba Scientific, XploRA plus, Montpellier, France). Chemical maps were obtained by a 532 nm laser as an excitation light with a 50% filter. The experimental conditions were as follows: 100 nm slit width, pinhole 100 µm, x50/0.90 NA Vis-LWD air objective, and 1 s acquisition time with 2 accumulations. The Raman signal was obtained using a 600 lines/mm grating centered between 800 and 3100 cm^−1^. The acquired spectra were corrected in a range from 1000 to 1800 cm^−1^, smoothed, and baseline corrected using LabSpec 6 Suite software. Each sample generated a cube of data with dimensions of 25 × 25 × 761, where 25 was the number of pixels at the x and y axes and 761 was the number of spectral variables.

### 2.4. Data Analysis of Chemical Maps

According to Vajna et al. [29], before chemometric evaluation, all spectra were baseline corrected (this was done by using the same baseline points for all maps and pure component spectra). Then, the spectral range from 1000 to 1800 cm^−1^ was used for the corresponding evaluation. Raman chemical map data were analyzed by using Solo+MIA software (Eigenvector, Research, Inc. Wenatchee, WA, USA). The raw 3-dimensional data were unfolded into a 2-dimensional matrix. The estimation of pure component spectra from the Raman chemical maps was carried out by MCR-ALS. This technique is based on the following bilinear model (Equation (1)):

(1) $$X=CS^{T}+E$$

where X (p∗λ) is the matrix containing the mapping spectra, $S^{T}$ (k∗λ) is the set of pure component spectra, and C (p∗k) contains the vectors of spectral concentrations (each row in C contains the concentrations of the k ingredients). The matrix E represents the residual noise. MCR–ALS generated both the concentration matrix C (scores) and recovered spectrum matrix $S^{T}$ (loadings) from the dataset X in an iterative manner, using an initial estimation for either C or $S^{T}$ and appropriate constraints.

#### Preprocessing and Constraints

The preprocessing technique was normalized (1-norm, area = 1). The normalization of concentration profiles or resolved spectra or use of reference concentration values within the optimization helps to suppress the rotational ambiguity [30]. The applied constraints were non-negativity, which forces the profiles to be formed by positive values and can be implemented replacing negative values by zeros [30], and equality, which makes the concentration profile and/or spectra of a component equal to a certain known predefined shape [30]. Pure CB, CNO, and SIO spectra were used as equality constraints.

### 2.5. Miscibility

To determinate the miscibility of the vegetable oils proposed as novel CBEs with CB, the homogeneity of the samples was quantitatively and qualitatively determined. The relative standard deviation (RSD) is a commonly used tool in the pharmaceutical industry to estimate the homogeneity of a component within a blend [31] and describe the distribution of the components quantitatively. The RSD was calculated from the ratio of the standard deviation (σ) and the mean (μ) of each measured image score. Using RMarkdown software, the RSD of the scores within a chemical image was calculated. A lower RSD of the chemical image corresponded to a more homogeneous distribution of the respective ingredient [29,32,33] and, therefore, its miscibility. Qualitatively, the homogeneity of the samples was analyzed using histograms. According to Gendrin et al. [33], a histogram showing a symmetric distribution with a narrow base and sharp peak is representative of a low-contrast image and therefore of a homogeneous sample. Conversely, an asymmetric histogram with a wide base and flatter peak or several modes is representative of a more contrasted image, i.e., a heterogeneous sample.

## 3. Results

### 3.1. Characterization of the Spectra of Cocoa Butter and Vegetable Oils

**Figure 1:**
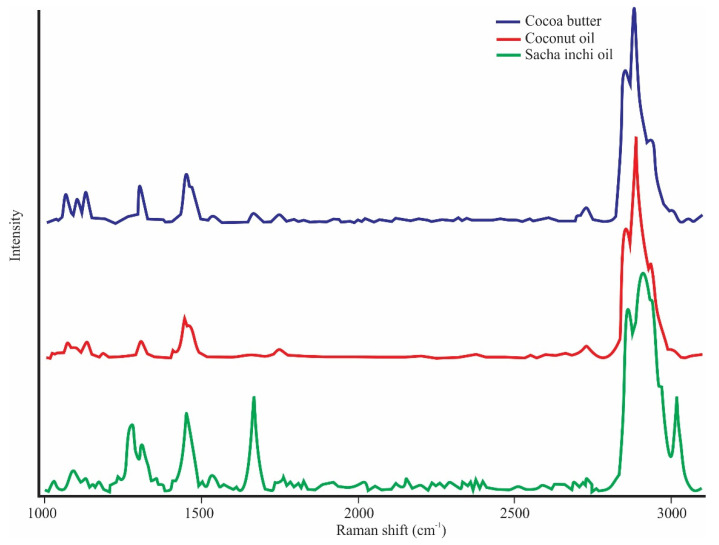
Raman spectra of the pure CB and vegetable oils in the full range (1000–3100 cm^−1^) at room temperature (20 °C).

**Figure 2:**
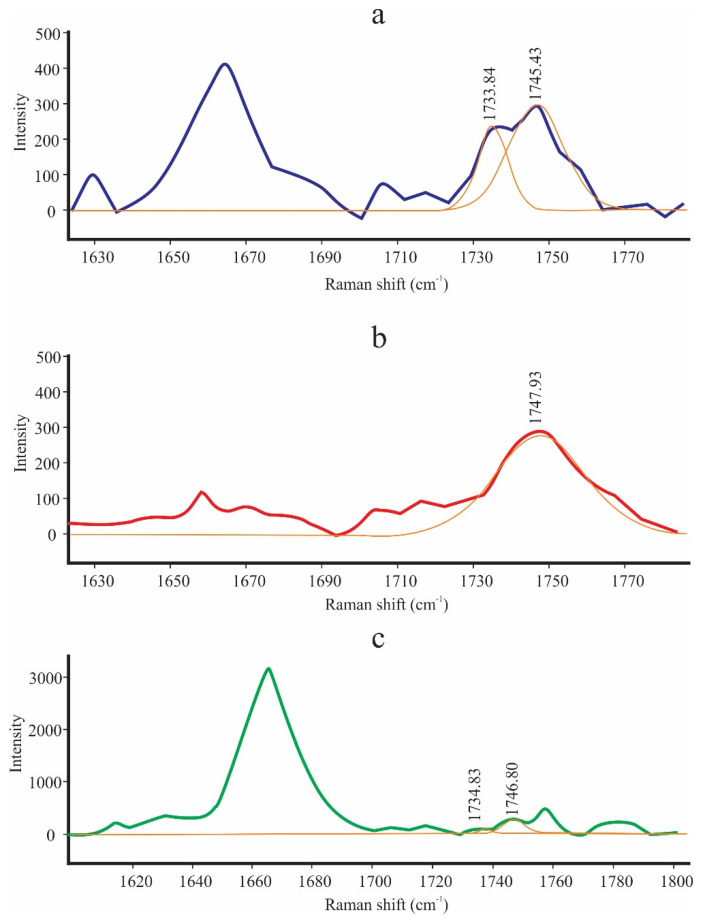
Carbonyl stretching region (1700–1780 cm^−1^) of CB and vegetable oils: (**a**) CB; (**b**) CNO; and (**c**) SIO.

Figure 1 shows the characteristics of the Raman spectra of the pure components (CB, CNO, and SIO) in the full range (1000–3100 cm^−1^) at room temperature (20 °C). In the CB spectra, the C–H stretching region shows peaks at 2885.7 and 2886.1 cm^−1^ which are assigned to alkyl-chain methylene symmetric (ν_s_) and antisymmetric (ν_as_) stretching. A peak at 2936.5 cm^−1^ associated with the terminal methyl ν_s_(CH_3_) stretch was observed. In the C=O stretching region, we can see 2 peaks at 1745.4 and 1733.8 cm^−1^, which are representative of forms III and IV at room temperature (Figure 2). The full width at half maximum (FWHM) of the peak at 1745.4 cm^−1^ was 17.38 cm^−1^. In the C=C stretching region [ν_s_(C=C)] of the olefinic band, we can see a peak at 1662.7 cm^−1^ (Table 2), representing the solid state of CB form IV at room temperature. This peak is of greater intensity in SIO due to its liquid state and is also related to the proportion of oleic acid. A total of 2 peaks at 1445.9 and 1462.9 cm^−1^ in the CH_2_ and CH_3_ deformation regions can also be seen. In the CH_2_ twisting region, we can also see a peak at 1301.3 cm^−1^, which is related to the degree of coupling of the alkyl chains in the lipids. The CB spectra also reveal 3 characteristic peaks of the CB polyforms, located in the C–C stretching range (1030–1183 cm^−1^). The peak at 1102.21 cm^−1^ indicates the existence of CB in its solid state at room temperature. This peak is not present in CNO and SIO. The pure spectra of CB, CNO, and SIO look similar (Figure 1); however, we can find some differences in some peaks. CNO has a peak at 1662.7 cm^−1^ whose intensity is lower. SIO has high intensity peaks at 1276.7 cm^−1^ and 3020.2 cm^−1^ that the others do not have. These peaks are assigned to plane =CH deformation in an unconjugated *cis* C=C and asymmetric C=H stretch group. From Figure 2, we can see that the peak at 1745.3 cm^−1^ in CB is shown at 1747.93 cm^−1^ in CNO and at 1746.8 in SIO, and the peak at 1733.84 cm^−1^ in CB is shown at 1734.83 cm^−1^ in SIO. We found differences between the area ratios of the peaks at 1733.84 and 1745.43 cm^−1^, which can be used as differentiation patterns between CB, CNO, and SIO.

Table 3 shows the area ratios and FWHM of CB, CNO, and SIO in the mode of vibrations ν(C=O). The lower FWHM values indicate a better arrangement of the crystals and their solid state; therefore, at room temperature, the crystals of CB (17.38 cm^−1^) would have a better arrangement than those of CNO (27.52 cm^−1^), demonstrating its solid state.

### 3.2. Miscibility of Cocoa Butter and Vegetable Oils

**Figure 3:**
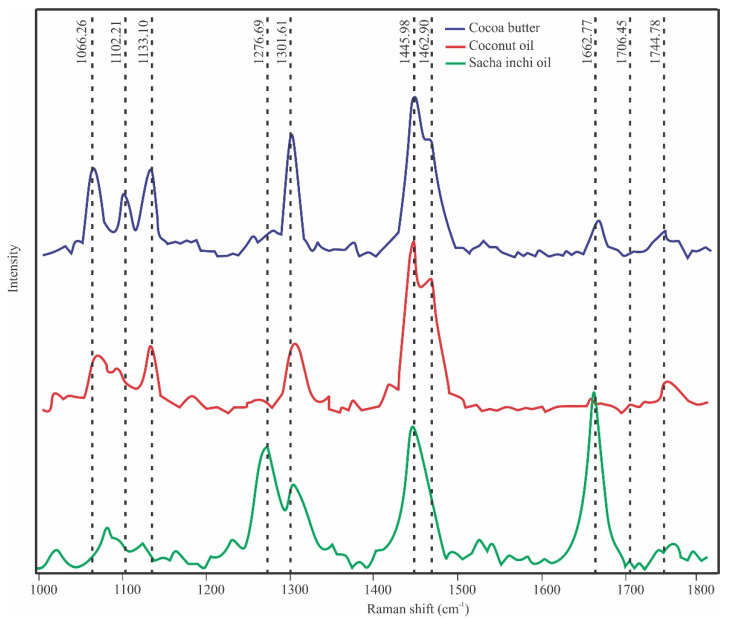
Raman spectral range used for analysis of the miscibility of CB and vegetable oils by MCR-ALS.

Figure 3 shows the spectral range used for the miscibility analysis between CB, CNO, and SIO. The Raman spectra of the pure components are similar due to the similarity in their chemical composition. However, there are important differences that are considered for the analysis by MCR–ALS. These differences are mainly found in the C–C stretching region (1000–1200 cm^−1^), the C=C stretching [ν_s_(C=C)], and the carbonyl C=O stretching region (1700–1800 cm^−1^). The peak located at 1662.77 cm^−1^ has a greater intensity in the SIO than in CB and CNO, and is characteristic of its liquid state.

**Figure 4:**
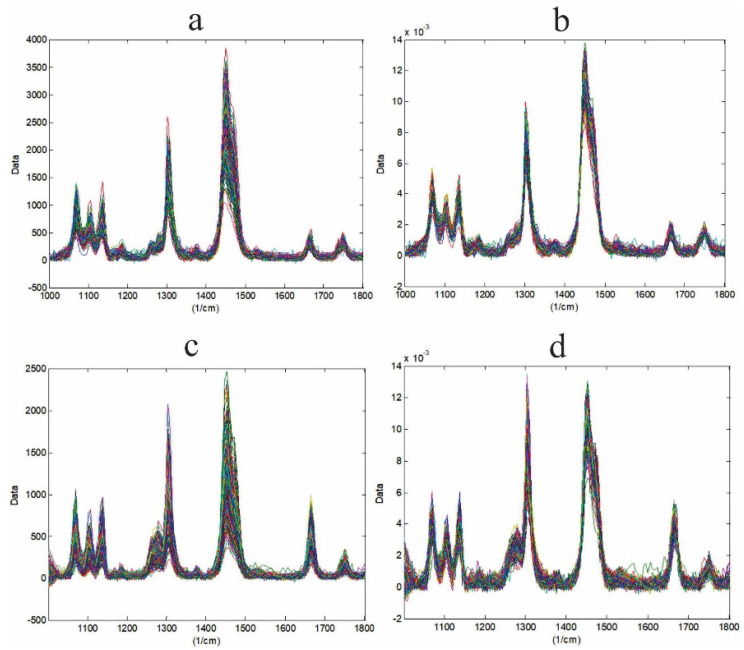
Raw data (**a**,**c**) and preprocessed data (**b**,**d**) from CB samples mixed with 25% CNO (**a**,**b**) and 25% SIO (**c**,**d**).

Figure 4 shows the effect of the 1-norm preprocessing technique on the raw data from samples CB75–CNO25 (Figure 4a) and CB75–SIO25 (Figure 4c) obtained by Solo + MIA (original data in Supplementary Material). This method was able to correct the noise and scattering contributions in the raw data (Figure 4b,d) before fitting the data to the MCR–ALS model.

Table 4 shows the MCR–ALS quality parameters and correlation coefficients between the original spectra and the spectra recovered ($S^{T}$) by MCR–ALS. The quality parameter related to the fit of the model was the percentage of explained variance, whose values were between 94.59 and 98.46%, acceptable for our work. The use of the spectra of the pure compounds as equality constraints produced correlation coefficients between 0.9999 and 0.9993. With these values, it can be verified that component 1 was related to CB and component 2 was related to vegetable oils (CNO or SIO) according to the analyzed samples.

**Figure 5:**
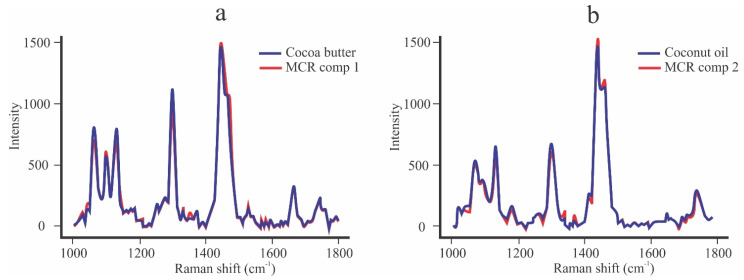
Comparison between the real spectra of the pure component and its respective spectrum recovered by MCR-ALS: (**a**) real and recovered spectra of CB; (**b**) real and recovered spectra of CNO.

Figure 5 shows a comparison between the spectrum recovered ($S^{T}$) by the MCR–ALS model and the original spectrum of the pure components. The restrictions used allowed for almost identical spectra with a good correlation. We can note that the spectrum of component 1 recovered by MCR–ALS is identical to the real spectra of CB (Figure 4a) and component 2 is identical to the real spectra of CNO (Figure 4b).

**Figure 6:**
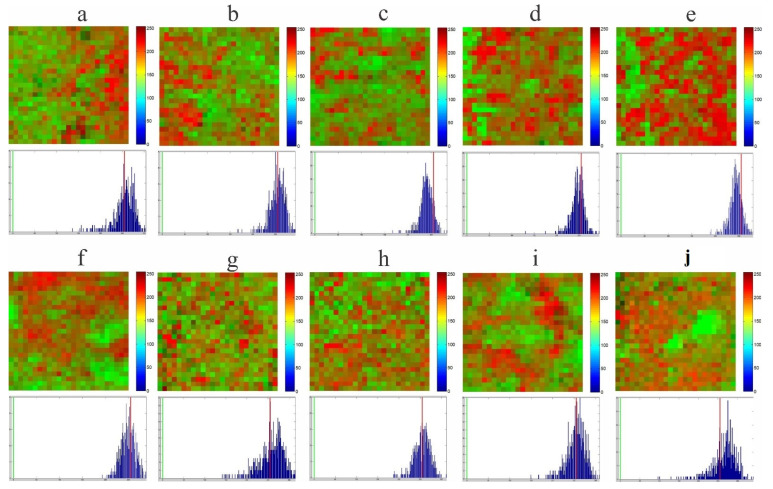
Distribution maps of the samples and their different concentrations: (**a**) CB55-CNO45; (**b**) CB65-CNO35; (**c**) CB75-CNO25; (**d**) CB85-CNO15; (**e**) CB95-CNO05; (**f**) CB55-SIO45; (**g**) CB65-SIO35; (**h**) CB75-SIO25; (**i**) CB85-SIO15; (**j**) CB95-SIO05.

Figure 6 shows the distribution maps and their corresponding histograms. These maps are constructed by the MCR–ALS model from the concentration matrix C and show the distribution of the compounds in the blend. The reddest areas correspond to higher concentrations and the greenest areas correspond to the lowest concentrations. We can note that, in general, the histograms corresponding to the distribution maps are peak-shaped and symmetric, which indicates that both the CNO (Figure 5a–e) and the SIO (Figure 5f–j) form a homogeneous blend with the CB. However, there are differences in the shapes between each histogram caused by the different concentrations of vegetable oil used in each sample.

Table 5 shows the quantitative analysis performed on the samples. The lowest RSD value of each component indicates its most homogeneous distribution in the sample. The RSD results show that CNO is more homogeneously distributed in the CB when its concentration is 45%, while its distribution is less homogeneous at 15% or 5%. The distribution of SIO in the sample is more homogeneous at 35% and less homogeneous at 15% or 5%. With these results, we can affirm that the CNO is more miscible with CB than SIO and that the miscibility of both oils improves by increasing their concentrations in the sample.

## 4. Discussion

### 4.1. Characterization of the Spectra of Cocoa Butter and Vegetable Oils

According to Carmona et al. [36], the spectra were examined separately in the wavenumber region from 1000 to 3100 cm^−1^ to find differences between them. Then, although the spectra for edible vegetable oils were similar (Figure 1), it could be seen that they exhibit some differences which are small but enable their discrimination [35]. Wang et al. [37] reported a peak at 3016 cm^−1^ in the Chinese-specific peony seed oil spectrum. This peak is located in the region =C–H stretching vibration of the methyl linoleate group (cis, cis diene) of RCH=CHR, and it is used in the evaluation of oils with different unsaturation degrees. The SIO spectrum has a peak at 3020.2 cm^−1^ (Figure 1, Table 2) that is not present in CB and CNO. This peak demonstrates the degree of unsaturation of the SIO. In the characterization of the Raman spectra of CB at 22 °C carried out by Bresson et al. [34], 2 peaks were reported at 1744 and 1732 cm^−1^, which are representative of forms III and IV. In the present work, these peaks were identified at 1745.4 and 1733.8 cm^−1^ (Figure 2, Table 2) and show that the existing conformational differences depend on the CB polymorphism. Bresson et al. [38] observed 3 components between 1750 and 1725 cm^−1^, 1730, 1735, and 1744 cm^−1^, which were attributed to the peak at 1735 cm^−1^ in CB or the peak at 1736 cm^−1^ in CBE for form V or VI. This peak was not observed in the CB, CNO, and SIO spectra (Figure 2, Table 3), so we can deduce that the V form was not present in the CB; therefore, the previous statement is corroborated.

In the olefinic band in the C=C stretching range (1200–1800 cm^−1^), Bresson et al. [34] attributed the liquid form of CB to the intensity of the peak located at ~1661 cm^−1^, as well as to the functional group present in oleic acid. That is, the higher intensity of this peak characterizes the liquid state of CB, and the lower intensity characterizes the solid state (form IV). This coincides with what is observed in Figure 2a,c, in which this peak is located at 1662.7 cm^−1^ in CB, CNO, and SIO. There is a noticeable difference in the intensity of this peak, since it is higher in the SIO, which determines its liquid state at room temperature and its proportion of oleic acid. Likewise, this peak was observed at 1658 cm^−1^ by De Géa Neves et al. [18] in the Raman spectrum of CNO and was used as a differentiating pattern between CNO and other vegetable oils. The peak at 1445 cm^−1^ is related to the C–H deformation vibration, and the peak at 1658 cm^−1^ is assigned to *cis* C=C bonds, both of which provide the degree of unsaturation value [35,37]. Therefore, these peaks can be useful to determine the degree of unsaturation of CB, CNO, and SIO.

In the CB spectra, the stretching C-C region (1030–1183 cm^−1^) allows its different polyforms to be identified. According to Bresson et al. [34], the existence of three peaks (1066.26, 1102.21, and 1133.10 cm^−1^) (Figure 3) at room temperature allows us to recognize that it is the IV or V form and the solid state of CB. The physical state of these ingredients must also be taken into account; that is, at room temperature, the semisolid CNO only presented a peak at 1133.10 cm^−1^, and the liquid SIO did not present any peak in this region. This characteristic of the SIO spectrum agrees with the spectra of peony seed oil, soybean oil, and extra virgin olive oil reported by Wang et al. [37]. Bresson et al. [34] affirms that the peak at 1100 cm^−1^ is representative of the solid state of CB and is not shown in the liquid state. This statement agrees with our results, since these peaks are not seen in CNO and SIO, which are not solid at room temperature.

To find differentiation patterns between CB and CBE, Bresson et al. [38] identified peaks at 1744, 1735, and 1730 cm^−1^ and found notable differences between the area ratios of these peaks. The results in Table 3 indicate that only two peaks were identified at 1733.84 and 1745.43 cm^−1^, which correspond to the peaks mentioned above. The area ratios of CB and SIO are very different, making their differentiation possible. It was not possible to calculate the area ratio for the CNO because the peak at 1733.84 cm^−1^ was not found. FWHM is also an indicator of polymorphism in the sense that a decrease in this value indicates the transition of CB from a liquid to a solid due to the better arrangement of the crystals [34]. This statement agrees with the results of Table 3, since the semisolid state of CNO will produce a higher FWHM than CB, which is solid at room temperature. The same behavior does not occur with SIO, since this vegetable oil is completely liquid at room temperature.

### 4.2. Miscibility of Cocoa Butter and Vegetable Oils

Following Mitsutake et al. [22], to start the analysis by MCR–ALS, the Raman range from 1000 to 1800 cm^−1^ was chosen, because it contains those peaks that allow differences to be found between the three materials studied. Therefore, Figure 3 shows the range of analysis and the main peaks that differentiate CB from CNO and SIO. The most striking difference is the high intensity of the peak of the SIO spectrum located at 1662.77 cm, which is related to its liquid state at room temperature. On the other hand, De Géa Neves et al. [18] reported the existence of peaks at 1264 and 1658 cm^−1^ in the CNO spectrum that differentiated it from other vegetable oils. In the present work, these peaks were shown at 1268.8 and 1662.2 cm^−1^, and their intensity was very low with respect to CB and SIO. According to Castro et al. [39], to remove noise signals and optimize the results of the MCR–ALS model, the data were preprocessed using the Savitzky–Golay filter from LabSpec 6 and the 1-norm normalization method from Solo + MIA. Figure 4b,d shows the preprocessed data showing low scattering contributions, with which they were corrected, obtaining acceptable results according to Zhang et al. [39,40].

The analysis of mixtures has been a constant concern in any scientific domain [41]. MCR–ALS solves this problem by providing a chemically (scientifically) significant additive bi-linear model of pure contributions from an original data matrix [30]. This bilinear model could produce several solutions, known as rotational ambiguity, which is the primary source of uncertainty. Thus, selecting the appropriate constraints is essential to obtain optimal solutions [30,41,42]. Once the optimization process has been finished, the MCR–ALS results are the set of concentration profiles, spectra and quality parameters (explained variance) related to the model [30]. Therefore, nonnegativity and equality restrictions were applied to our data. With these considerations, the explained variance of the MCR–ALS model for all samples was between 94.59 and 98.46% (Table 4), which means that this model is capable of representing the original data with high precision. Mitsusake et al. [22] reported explained variance percentages between 98.9 and 99.6% in MCR–ALS when it was applied to blends formulated using natural excipients, and Zhang et al. [40] reported values between 99.43 and 99.56% in the analysis of the constituents of commercial chocolate samples. The authors conclude that their results are well adjusted, and therefore, the MCR–ALS model is capable of constructing chemical maps of the samples. Although these values are higher than the results found in our work, we can say that our data fit the MCR–ALS model in such percentages.

Zhang et al. [40] worked with white chocolates, making a comparison between the spectrum of the pure components such as sucrose, lactose, butter and whey. The correlation coefficients between the pure spectra and those recovered by MCR–ALS with data preprocessing were between 0.6701 and 0.9910, which were considered satisfactory. The equality constraint fixes the recovered spectra or concentrations to specific known values [43]. This is the reason why the values of the correlation coefficient between the recovered spectra and the pure compounds are high (Table 4). Additionally, Figure 5 shows that there is no rotational ambiguity, because the recovered spectra ($S^{T}$) are identical to the original spectra. The same results were obtained for the other samples. Based on these results, we can affirm that the first spectrum recovered by MCR–ALS (component 1) corresponds to CB, while the second spectrum (component 2) corresponds to vegetable oil, according to the sample analyzed.

The mathematical analysis of each image allows for the extraction of parameters that are helpful in the interpretation of the images and in understanding of the blending process studied [31]. The data provided by Raman mapping contain spectral and spatial information; then, MCR–ALS can be used to visualize the concentration distribution maps of the different components present in a sample based on their individual spectral signals [44]. However, the quality of the Raman mapping is limited by the spectra of the individual compounds and their concentration, so the analysis becomes complex if the spectra of the components have common peaks [21], as is the case with CB, CNO and SIO (Figure 3). This was another reason why we decided to use the spectra of the pure compounds as equality constraints. It is important to mention that the values in matrix C are related to the concentrations, but they are not the real concentrations of the components of the blends, so the mean value should not be compared with the real concentration of each component [22]. Figure 6 shows the distribution maps of CB, CNO, and SIO in all the samples analyzed constructed from the matrix of concentrations C obtained by MCR–ALS. Figure 6a shows a better distribution of CB and CNO at concentrations of 55 and 45%, respectively. Similarly, Figure 6g shows a better distribution of CB and SIO at concentrations of 65 and 35%, respectively. Both figures show better distribution than the others. Similar results were obtained by Scoutaris et al. [21] when analyzing mixtures of paracetamol (PMOL) and compritol 888 (C-888).

We consider that the miscibility of two components can be determined by their distribution in a chemical map, and homogeneous distribution is an indicator of good miscibility. The homogeneity of the samples is also analyzed using the histograms of the distribution map. According to Gendrin et al. [45], a histogram that exhibits a symmetric distribution with a narrow base and a sharp peak is representative of an image with a low contrast, and therefore a homogeneous sample. The histograms corresponding to each distribution map show a symmetric shape in all cases (Figure 6), with differences according to the actual concentration of each sample. Lyon et al. [46] prepared tablets composed of furosemide and excipients at five different degrees of mixing, reporting that the most homogeneous distribution was obtained in those samples whose histograms were symmetric.

The homogeneity of the samples can be quantitatively analyzed using the RSD. According to Scoutaris et al. [21], RSD has been used to compare the homogeneity of a sample; a low RSD value is interpreted as signifying higher homogeneity. Mitsusake et al. [22] showed that the standard deviation of histograms (STD) is used to evaluate the miscibility for the preformulation stage of pharmaceutical tablets. Therefore, we consider that both parameters are comparable. Lyon et al. [46] used the RSD of the histograms generated by the image scores to determine the homogeneity of the distribution of furosemide in tablets, observing a progressive increase in RSD as the degree of homogeneity decreased. Mitsutake et al. [22] observed an intermediate miscibility (STD = 6.9) between CB and CNO at real concentrations of 75 and 25%, respectively. Similar results were obtained in the present study (Table 5), in which it is shown that blends containing 45% CNO and 35% SIO have the lowest RSD; therefore, they form a more homogeneous blend with CB and are more miscible at those concentrations. In the elaboration of CBEs, the candidate vegetable oil must have an SOS triglyceride concentration similar to that of CB. To achieve this, it undergoes a fractionation process [8]. In the case of CNO and SIO, they were used in their natural state, showing good miscibility with CB; therefore, they would be good candidates to be used as CBE.

Food products are complex mixtures of heterogeneous nature; obtaining chemical and spatial information from them is crucial for food safety and quality control [47]. For this, food detection technologies play a fundamental role [25]; therefore, it is urgent to develop rapid methods of nondestructive analysis to control the quality and safety of food and thus control its circulation in the market [47]. Chemical Raman imaging (CRI), in combination with chemometrics, can provide spectral information and spatial distributions of specific chemicals, analyzing them non-destructively [42,47,48,49,50]. However, in the food field, only a few investigations on the application of CRI have been reported [50]. CRM allows the application of Raman mapping with MCR-ALS to obtain the chemical characteristics of CB, CNO, and SIO through their Raman spectral fingerprint (Figure 1) and to identify the miscibility of these three components (Figure 6, Table 5), which demonstrates the usefulness of this methodology in initiating the development of new products in the chocolate industry. Some authors have also used this methodology, such as Liu et al. [51], who used chemical Raman mapping to study the compatibility between hydroxypropyl methylcellulose (HPMC) and gelatin. It was found that HPMC was easily adapted to form continuous and intermediate phases from the molecular interactions between both components. Mitsutake et al. [52] studied the miscibility and structural changes (polymorphism) in mixtures of natural and synthetic beeswax (BW) with copaiba oil using Raman mapping and MCR-ALS. Structural changes were found in the synthetic BWs, and the miscibility between both BWs with copaiba oil was not significantly different. It was also observed that the differences between the freshly prepared mixtures and those with three months of storage were more significant when the amount of oil was increased. On the other hand, Rodríguez et al. [12] studied the compatibility of shea butter and CB mixtures using the isosolid diagram but did not use Raman mapping.

Lauric acid and partially hydrogenated fats are not recommended in the chocolate industry because they can increase LDL cholesterol levels [53]. Norazlina et al. [2] reported the following CBE candidates: mean fraction of palm oil, mango seed fat, shea stearin, kokum fat, illipe butter, high oleic and stearic sunflower oil, palm stearin, and bambangan kernel fat. However, this author does not report CNO and SIO. The results of the present work show CNO and SIO as possible candidates for novel CBE, as they demonstrate some advantages, such as their high degree of unsaturation, which makes them healthy fats, as well as their excellent molecular compatibility with CB demonstrated by their miscibility. However, it is necessary to carry out some additional studies, such as the investigation of their thermal and rheological properties at different concentrations of solid and liquid lipids and the investigation of their behaviors over time.

## 5. Conclusions

In the present work, the usefulness of the confocal Raman microscopy (CRM) technique to identify the chemical properties of cocoa butter, coconut oil and sacha inchi oil is demonstrated. These latter vegetable oils are proposed as candidates to be cocoa butter equivalents in the manufacture of chocolates. The main differences are in the physical state and the degree of unsaturation, which are differentiated by the intensity of the peaks in the Raman spectra. Likewise, the usefulness of the chemometric technique known as multivariate curve resolution–alternating least squares to analyze the miscibility of these vegetable oils with cocoa butter is demonstrated. We conclude that coconut oil is more miscible with cocoa butter at a 45% concentration, and sacha inchi oil is more miscible at a 35% concentration. Between the two vegetable oils, coconut oil is more miscible than sacha inchi oil. We consider that this work is the first step in finding novel CBEs for developing new chocolates. Further work is necessary to evaluate their thermal, rheological, and sensorial properties.

## Figures and Tables

**Table 1:** Composition of the samples.

Sample	Cocoa Butter (%)	Coconut Oil (%)	Sacha Inchi Oil (%)
CB55-CNO45	55	45	---
CB65-CNO35	65	35	---
CB75-CNO25	75	25	---
CB85-CNO15	85	15	---
CB95-CNO05	95	05	---
CB55-SIO45	55	---	45
CB65-SIO35	65	---	35
CB75-SIO25	75	---	25
CB85-SIO15	85	---	15
CB95-SIO05	95	---	05

**Table 2:** Raman peaks for CB and vegetable oils.

Assignments ^1^	Cocoa Butter (cm^−1^)	Coconut Oil (cm^−1^)	Sacha Inchi Oil (cm^−1^)
ν_as_(C–C)T	1066.2	1069.3	Nd
ν(C–C)G	1102.9	1092.4	Nd
ν_s_(C–C)_T_	1132.3	1132.3	1125.7
τ(CH_2_)	Nd	1268.8	Nd
τ(CH_2_)	Nd	Nd	1276.7
τ(CH_2_)	1301.3	1303.4	1308.8
δ(CH_2_)	1445.9	1445.1	1449.9
δ_a_(CH_3_)	1462.9	Nd	Nd
ν_s_(C=C)	1662.3	1662.2	1662.7
ν(C=O)	1733.8	Nd	1734.8
ν(C=O)	1745.4	1747.9	1746.8
ν(CH_3_–CH_2_)	2728.7	2730.8	2733.9
ν_s_(CH_2_)	2855.7	2856.7	2863.4
ν_as_(CH_2_)	2886.1	2886.1	2907.6
ν_s_(CH_3_)	2936.5	2932.3	Nd
(=CH)^2^	Nd	Nd	3020.2

^1^ Assignments according to Bresson et al. [34], and Jiménez-Sanchidrián et al. [35].

**Table 3:** FWHM and area ratios of the components of the Gaussian function of Raman spectra of CB, CNO, and SIO at room temperature (T = 20 °C).

Component	1733.84 cm^−1^		1745.43 cm^−1^		Area Ratio
	Area	FWHM	Area	FWHM	A_1733.84_/A_1745.43_
Sacha inchi oil	288.19	3.99	2751.58	9.04	0.11
Coconut oil	Nd	Nd	8112.33	27.52	Nd
Cocoa butter	2448.96	9.85	5366.85	17.38	0.46

**Table 4:** MCR–ALS quality results and correlation coefficients between recovered spectra by the model and real spectra.

Sample	Numbers of Factor	Explained Variance (%)	MCR-ALS Component	Cocoa Butter	Vegetable Oil
CB55-CNO45	2	96.74	Comp 1	0.9999	0.9396
			Comp 2	0.9384	0.9999
CB65-CNO35	2	96.60	Comp 1	0.9997	0.9546
			Comp 2	0.9397	0.9996
CB75-CNO25	2	98.14	Comp 1	0.9998	0.9378
			Comp 2	0.9401	0.9999
CB85-CNO15	2	98.46	Comp 1	0.9996	0.9377
			Comp 2	0.9408	0.9999
CB95-CNO05	2	92.76	Comp 1	0.9999	0.9386
			Comp 2	0.9404	0.9998
CB55-SIO45	2	94.59	Comp 1	0.9998	0.5944
			Comp 2	0.6117	0.9993
CB65-SIO35	2	97.46	Comp 1	0.9999	0.5976
			Comp 2	0.6099	0.9995
CB75-SIO25	2	97.99	Comp 1	0.9998	0.9995
			Comp 2	0.6121	0.5976
CB85-SIO15	2	98.01	Comp 1	0.9999	0.5924
			Comp 2	0.6147	0.9993
CB95-SIO05	2	97.39	Comp 1	0.9997	0.5914
			Comp 2	0.6140	0.9995

**Table 5:** Miscibility of vegetable oils with cocoa butter at different concentrations determined by their RSD.

Sample	Cocoa Butter RSD ^1^	Vegetable Oil RSD ^1^
CB55-CNO45	0.12 ± 0.01 ^ab^	0.09 ± 0.02 ^b^
CB65-CNO35	0.17 ± 0.03 ^ab^	0.21 ± 0.09 ^ab^
CB75-CNO25	0.23 ± 0.06 ^a^	0.29 ± 0.09 ^ab^
CB85-CNO15	0.21 ± 0.12 ^a^	0.47 ± 0.13 ^a^
CB95-CNO05	0.18 ± 0.04 ^ab^	0.44 ± 0.23 ^a^
CB55-SIO45	0.12 ± 0.02 ^ab^	0.25 ± 0.03 ^ab^
CB65-SIO35	0.10 ± 0.01 ^ab^	0.15 ± 0.04 ^b^
CB75-SIO25	0.10 ± 0.04 ^ab^	0.18 ± 0.04 ^ab^
CB85-SIO15	0.07 ± 0.01 ^b^	0.24 ± 0.03 ^ab^
CB95-SIO05	0.07 ± 0.02 ^b^	0.19 ± 0.03 ^ab^

^1^ Different letters (a,b) in the same column represent significant differences (*p* ≤ 0.05).

## Data Availability

Not applicable.

## Supplementary Materials

The following are available online at https://www.mdpi.com/article/10.3390/foods10123101/s1, map blend: CB–CNO; CB–SIO and pure spectrum: CB; CNO; SIO.
